# Tailoring the Spacer Arm for Covalent Immobilization of *Candida antarctica* Lipase B—Thermal Stabilization by Bisepoxide-Activated Aminoalkyl Resins in Continuous-Flow Reactors

**DOI:** 10.3390/molecules21060767

**Published:** 2016-06-13

**Authors:** Emese Abaházi, Dávid Lestál, Zoltán Boros, László Poppe

**Affiliations:** 1 Department of Organic Chemistry and Technology, Budapest University of Technology and Economics, Műegyetem rkp. 3, Budapest H-1111, Hungary; abahazi.emese@mail.bme.hu (E.A.); ldavka@gmail.com (D.L.); 2 SynBiocat LLC; Lövőház u. 19/1, Budapest H-1043, Hungary; zoltan.boros@synbiocat.com

**Keywords:** lipase, immobilization, covalent attachment, kinetic resolution, thermal stabilization, continuous-flow reactor

## Abstract

An efficient and easy-to-perform method was developed for immobilization of *Ca*LB on mesoporous aminoalkyl polymer supports by bisepoxide activation. Polyacrylate resins (100–300 µm; ~50 nm pores) with different aminoalkyl functional groups (ethylamine: EA and hexylamine: HA) were modified with bisepoxides differing in the length, rigidity and hydrophobicity of the units linking the two epoxy functions. After immobilization, the different *Ca*LB preparations were evaluated using the lipase-catalyzed kinetic resolution (KR) of racemic 1-phenylethanol (*rac*-**1**) in batch mode and in a continuous-flow reactor as well. Catalytic activity, enantiomer selectivity, recyclability, and the mechanical and long-term stability of *Ca*LB immobilized on the various supports were tested. The most active *Ca*LB preparation (on HA-resin activated with 1,6-hexanediol diglycidyl ether—HDGE) retained 90% of its initial activity after 13 consecutive reaction cycles or after 12 month of storage at 4 °C. The specific rate (*r*_flow_), enantiomer selectivity (*E*) and enantiomeric excess (*ee*) achievable with the best immobilized *Ca*LB preparations were studied as a function of temperature in kinetic resolution of *rac*-**1** performed in continuous-flow packed-bed bioreactors. The optimum temperature of the most active HA-HDGE *Ca*LB in continuous-flow mode was 60 °C. Although *Ca*LB immobilized on the glycerol diglycidyl ether (GDGE)-activated EA-resin was less active and less selective, a much higher optimum temperature (80 °C) was observed with this form in continuous-flow mode KR of *rac*-**1**.

## 1. Introduction

Lipases are ubiquitous enzymes which can be found in animals, plants, fungi and bacteria [1,2]. Naturally, lipases catalyze the hydrolysis of esters formed of glycerol and long-chain fatty acids [3,4]. However, under particular conditions, they can catalyze esterification and transesterification [5] with high activity, specificity and selectivity. Therefore, lipases represent a significant class of industrially relevant enzymes [6].

Lipases represent a peculiar mechanism of action, the so called interfacial activation [7,8]. Due to a loop containing α-helicoidal elements that covers the active site, a large proportion of lipases have two different conformations (the closed and the open forms) [4,9]. In an aqueous environment, the equilibrium is shifted towards the water soluble closed form and the active site is secluded from reaction medium [4,10,11]. Catalysis takes place in the open-lid form which can be stabilized by interfacial activation at the interface of the drops of the oily phase containing hydrophobic substrates [12]. These structural changes related to the interfacial activation are crucial to provide the substrates access to the active site. The performance of enzymes in organic solvents facilitated their application as biocatalysts [13], even in supercritical medium [14].

The lipase B from *Candida antarctica* (*Ca*LB) is one of the most frequently used lipase in biotechnology due to its excellent biochemical properties such as resistance to organic solvents, high stereo- and enantioselectivity [2,9,15]. The three-dimensional structure of *Ca*LB has been resolved by Uppenberg [16,17]. *Ca*LB—having a molecular weight of 33 kDa and isoelectric point of 6.8—contains only a small lid covering the active site [18]. Therefore, it was thought that the typical interfacial activation of lipases [7,8] is not a characteristic feature of this enzyme [19]. However, recent structural investigations revealed the open and closed states of *Ca*LB and the mechanism of interfacial activation [20].

Replacing soluble enzymes with immobilized preparations in synthetic processes can significantly reduce the cost of enzyme production and complex downstream processing [21,22]. Immobilization of enzymes is a powerful biochemical tool with a potential for improving enzyme activity, temperature stability, and selectivity [23]. Retaining the active conformation during immobilization is very important for enzyme activity [24]; therefore, finding an immobilization protocol that increases enzyme activity and stability should be a target of real importance [25]. Several immobilization techniques are available for protein immobilization: adsorption, covalent attachment onto solid supports, cross-linking and entrapment of protein in matrices. Generally, a support for enzyme immobilization must meet two requirements: it should contain an adequate amount of functional groups on the surface and it should be mechanically stable for repeated use or in application in continuous processes [21]. Adsorption of lipases on hydrophobic surfaces is easy to perform because they resemble the surface of their natural substrates [4], but enzyme leaching could be a serious disadvantage [22,26]. After adsorption on the hydrophobic surface, post-immobilization with glutaraldehyde (GA) is frequently used [2]. GA modifies primary amino groups of proteins and it is also an effective cross linker used to produce CLEAs (Cross-linked enzyme aggregate) [27]. GA can be used for activating the primary amino groups of the support followed by covalent immobilization [3,25]. Entrapment could be a proper immobilization method for enzymes which can be easily deactivated as in this way the three dimensional structure of enzyme could be stabilized via rigidification by the entrapping matrix [28].

In the past few decades, intensive studies were performed on development of supports for lipase immobilization. The most straightforward strategy was to utilize hydrophobic supports for lipase immobilization in their active conformation by simple adsorption advancing interfacial activation. Hydrophobic organic materials such as octyl-sepharose or octadecyl-polyacrylate [4] and polyethyleneimine-agarose [29] were successfully applied in adsorptive immobilization where lipase stayed fully active even after a very long incubation in organic solvents. Polymer supports with core-shell morphology could tune lipase properties during immobilization [12]. Similarly, surface-grafted mesoporous silica gel as inorganic material proved to beneficial for adsorptive immobilization of lipases [2,30,31,32].

Although lipases after adsorptive immobilization can retain their activity in organic media but under aqueous conditions they can release the enzyme causing decrease of activity and product contamination. To circumvent the problem of enzyme leaching, covalent immobilization offered a solution. For example, activated natural polymers (epichlorohydrin- or glutaraldehyde-activated chitosan or agarose gels) proved to be suitable carriers for multipoint covalent immobilization of proteins [3,33] or silica gel grafted with glyoxyl groups enabled covalent enzyme immobilization [34].

Epoxy or multifunctional epoxy-supports were suitable for covalent immobilization of enzymes [32,35,36]. Epoxy supports—while being stable at neutral pH values for a long time in wide temperature range—could react with different nucleophilic groups (such as amine, thiol or carboxylate) on the protein surface under mild conditions by forming stable covalent bonds which prevents enzyme leakage [21,37,38]. The usefulness of epoxy-functionalized carriers [33,34,35,36,37,38,39] led to the idea of using bisepoxides for enzyme immobilization. Thus, poly(ethylene glycol) diglycidyl ether was applied for binding various oxidases onto biosensor microelectrode [40]. Later, glycerol diglycidyl ether was used as a cross-linking agent for immobilization of lipases and phenylalanine ammonia-lyase (PAL) as cross-linked enzyme aggregates (CLEA) [41] or binding enzymes such as PAL [42] or *Ca*LB [43] onto carbon nanotubes. 

Besides enzyme immobilization, reactor technology can also affect the efficiency of biotransformations [44]. Nowadays, continuous-flow systems are of ever-increasing importance since they offer facile automation, reproducibility and safety [45,46]. In addition to the ease of studies on substrate concentration, temperature and flow rate and how these factors effect enzyme-catalyzed reactions in such systems, the catalytic efficiency of the immobilized enzymes proved to be higher in packed-bed reactors operated in continuous-flow mode than in batch mode [47,48,49]. Noteworthy, the mechanical damages reducing the operational stability of the immobilized biocatalysts in stirred or shaken batch reactors is much less pronounced in packed bed reactors. The influence of various immobilization conditions [48,50] and of ionic liquids [51] on biocatalytic properties of *Ca*LB was already evaluated in continuous-flow processes.

The aim of this study was to investigate the activation of mesoporous aminoalkyl polymer resins using low-cost bisepoxides in covalent immobilization of lipase B from *Candida antarctica* (*Ca*LB) offering a less-toxic, inexpensive and easy-to-handle alternative over glutaraldehyde-activation of such carriers. A major concern was to fine-tune the length, flexibility and hydrophobicity of the spacer arm by varying aminoalkyl functions and bisepoxides, thereby resulting in significant variation in the properties of the *Ca*LB biocatalysts by altering the microenvironment surrounding the enzyme molecule during and after immobilization. Variation of the spacer arm offers tuning and optimization possibilities in biocatalyst design and development and can lead to improved properties useful for extended use in recycled batch reactions or in continuous-flow systems even at elevated temperatures.

## 2. Results and Discussion

Selection of the supports was a crucial point for this study on surface fine-tuning exploring variations created by different bisepoxide spacer arms. Besides the functions on the surface, pore size and particle size were important parameters influencing the final properties of resins in various applications.

### 2.1. Selection and Bisepoxide Activation of Carriers Used for Covalent Immobilization of CaLB

Because a final goal was to apply the immobilized *Ca*LB derivatives in continuous-flow reactors, particle size influencing pressure drop and pore diameter affecting specific area for immobilization and mass transfer properties were important parameters. Thus, two commercially available macroporous polymethyl methacrylate enzyme carriers functionalized with ethylamine (EA) and hexylamine (HA) at their surface were selected as starting supports for this study. The properties of the resins and approximate density of ethylamine and hexylamine functional groups on resin surfaces are presented in Table 1. Worth mentioning is that the different lengths of ethylamine and hexylamine functional groups on the two starting carriers can contribute to the different properties of the final spacer arms between the surface of carriers and the target protein.

### 2.2. Covalent Immobilization of CaLB on Bisepoxide-Activated Supports and Its Properties in Kinetic Resolution of 1-Phenylethanol rac-**1**

**Figure 1:**
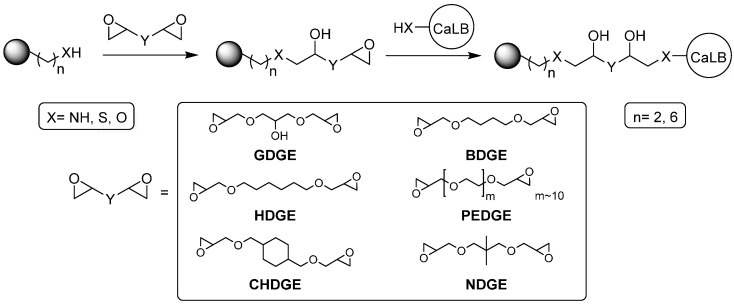
Bisepoxide activation of supports for immobilization of *Ca*LB.

Due to their quite high content of water (~70%), drying of the resins was required to determine their real mass before surface modification (see Section 3.3.). In the first set of experiments, six different types of bisepoxides with different lengths and hydrophobicities were selected as activating agents for the two aminoalkyl carriers (Figure 1). As reference, glutaraldehyde activation of the two carriers was also performed. After pre-activation, immobilization of *Ca*LB was carried out in phosphate buffer (pH 7.5) at room temperature (24 h).

Because the epoxide functions may form covalent bonds not only via the surface exposed amine functions of Lys residues but also via the S- and O-atoms of Cys, Tyr, Asp, Glu, covalent bond formation probability may be higher with the bisepoxide-activated supports than with the GA-activated ones, especially in the case of proteins having only a few surface exposed lysine residues. These differences may explain the higher activity of the immobilized *Ca*LB achieved with several bisepoxide-activated carriers compared to the GA-activated ones. Moreover, the C−X bonds (X = NH, S, O) forming by immobilization with the bisepoxide-activated carriers, unlike the C=N bonds forming in GA-activated ones, are not susceptible to hydrolysis.

Worth mentioning is that GA-activated resins are not suitable for long-term storage and they should be used for immobilization shortly after activation. On the other hand, the bisepoxide-activated supports—similarly to the usual epoxy carriers—can be stored for a long time enabling real separation of the carrier activation and enzyme immobilization steps in time.

**Figure 2:**
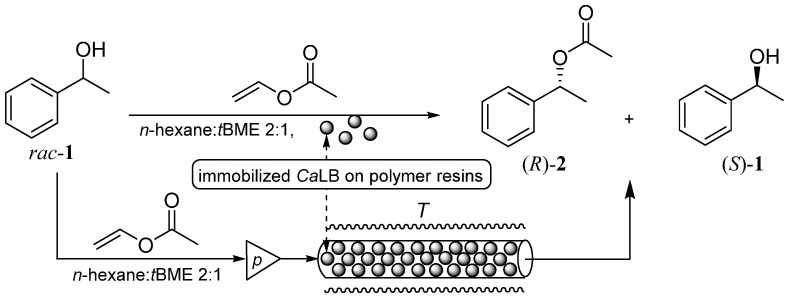
Kinetic resolution of racemic 1-phenylethanol (*rac*-**1**) catalyzed by covalently immobilized *Ca*LB preparations in batch mode and in continuous-flow reactor (for details of experiments see Section 3.7 and Section 3.12).

To characterize the catalytic performance of the biocatalysts, kinetic resolution of 1-phenylethanol (*rac*-**1**) was selected as the test reaction (Figure 2). This test enabled gaining information on the activity and enantiomer selectivity of the *Ca*LB variants. To distinguish between the fraction of *Ca*LB retained by only physical adsorption from the one retained by stronger covalent bonds, the activity of the preparations was tested before and after washing with Triton X-100 non-ionic detergent solution which could remove enzyme molecules attached only by hydrophobic interactions onto the surface. Table 2 shows the wash resistance representing the covalent ratio of binding onto activated EA- and HA-resins and the biocatalytic performance of the covalently bound *Ca*LB preparations.

For comparison, a commercial preparation (EP *Ca*LB) was selected as a standard in which *Ca*LB was covalently attached to macroporous acrylic beads of 150–300 µm particle size by the aid of epoxy functions (EP). In the case of EP *Ca*LB, washing the preparation with Triton X-100 solution resulted in 125% activation possibly due to a slight bioimprinting effect [53]. In case of EA *Ca*LB, the 7% wash resistance was consistent with the fact that the non-activated resin did not contain groups for covalent attachment and only ionic interactions could retain the enzyme molecules causing residual activity. In case of HA *Ca*LB, the alkyl chain of hexylamine function rendered the surface more hydrophobic compared to EA-resin, thus a combination of the increased hydrophobicity and ionic interactions could rationalize the slightly higher wash resistance (11%).

When polyethylene diglycidyl ether (PEDGE) was used as activating bisepoxide, the wash resistance remained quite similar as observed for the unmodified resins (7% for EA-PEDGE *Ca*LB and 17% for EA-PEDGE *Ca*LB). This result indicating good adsorption ability but only occasional covalent bond formation could be explained by assuming that during the activation both epoxy functions of the bisepoxide with a long spacer arm of high flexibility could react with the surface amine functions. Thus, only a few epoxy functions of PEDGE remained intact after the activation step explaining the low frequency of covalent bond formation of the PEDGE-activated resin with the enzyme.

In contrast, high wash resistance was obtained with *Ca*LB immobilized on 1,4-cyclohexanedimethanol diglycidyl ether (CHDGE)-, 1,6-hexanediol diglycidyl ether (HDGE)- and glycerol diglycidyl ether (GDGE)-activated resins (81%, 58% and 53%, respectively). This result can be attributed to the hydrophobic character of CHDGE, since cyclohexyl rings increased the hydrophobicity of support. Barbosa *et al.* proved that proteins become immobilized on epoxy supports via a two-step mechanism: physical adsorption occurs in the first place then covalent linkages are formed between the nucleophilic residues on the enzyme molecule and epoxy functions of the support [15]. Thus, enhanced hydrophobicity of the spacer arm with cyclohexyl ring could result in stronger hydrophobic activation and consequently lead to *Ca*LB forms of higher activity. A comparison of the activation of the alkylamino resins with bisepoxides to the widely applied glutaraldehyde activation indicated the value of the activation by a properly selected bisepoxide. The usefulness of a bisepoxide-activation-based approach was indicated clearly by the approximately four-fold activity of the *Ca*LB attached to CHDGE-activated supports (*U*_b_ = 39.6 µmol·min^−1^·g^−1^ for EA-CHDGE *Ca*LB and *U*_b_ = 47.6 µmol·min^−1^·g^−1^ for HA-CHDGE *Ca*LB) compared to the activity of the corresponding *Ca*LB form immobilized by the aid of the widely used glutaraldehyde-activation (*U*_b_ = 10.2 µmol·min^−1^·g^−1^ for EA-GA *Ca*LB and *U*_b_ = 11.9 µmol·min^−1^·g^−1^ for HA-GA *Ca*LB). The importance of the length of spacer arms became apparent by analysis of the differences between bisepoxide-activation by various bisepoxides. Within both series (EA-resin-based or HA-resin-based), the highest degree of covalent immobilization (wash resistance) was achieved after CHDGE activation (92% with HA-resin and 81% with EA-resin). HDGE and GDGE activation resulted in a somewhat lower degree of covalent binding (82% and 64% with HA-resin and 58% and 53% with EA-resin, respectively). However, only a modest degree of covalent binding was observed after BDGE-activation (51% with HA-resin and 22% with EA-resin). 

Analysis of the activities of the various forms of *Ca*LB covalently immobilized on pre-activated resins indicated a role of the alkylamino function of the resins influencing the properties of immobilized *Ca*LB. Residual activity of *Ca*LB attached to the modified HA-resins after activation with any bisepoxide or glutaraldehyde was higher than that attached to the corresponding pre-activated EA-resin. Because the trends within the two series were the same, optimization of the bisepoxide-activation conditions for *Ca*LB immobilization on either alkylamino resins was performed only with the three best bisepoxides (CHDGE, HDGE and GDGE).

### 2.3. Optimization of the Bisepoxide Activation for Covalent Immobilization of CaLB

**Figure 3:**
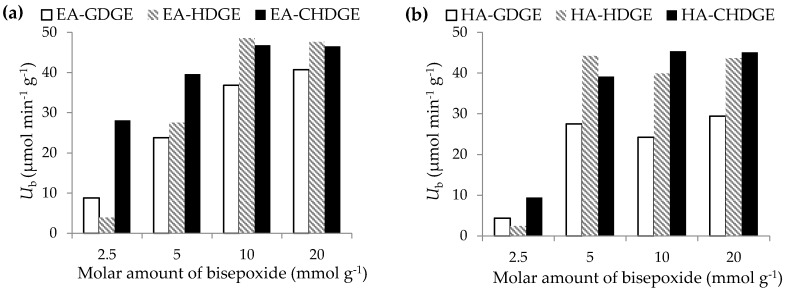
Optimization of the bisepoxide activation of ethylamine (EA) and hexylamine (HA) resins for *Ca*LB immobilization. Effect of molar amount of bisepoxide to mass resin ratio (mmol·g^−1^) of CHDGE, HDGE and GDGE on *Ca*LB activity. Experiments were performed as described in Section 3.7.

The microenvironment of enzymes after immobilization can be tuned by proper optimization of the process parameters. Our preliminary results applying bisepoxides in 5 mmol·g^−1^ of carrier amounts showed (Table 2) that aminoalkyl (EA or HA)-resin-activation by three bisepoxides were efficient for immobilization of *Ca*LB. In this series of experiments, the amount of bisepoxides applied for surface modification of the two aminoalkyl resins was varied in order to investigate the effect of final epoxy group density on enzyme activity and immobilization yield (Figure 3). Reduction of the amount of bisepoxides to 2.5 mmol·g^−1^ carrier caused dramatic decrease in specific activity of EA-HDGE *Ca*LB and EA-GDGE *Ca*LB. Increase of the molar amount of bisepoxides from 5 mmol·g^−1^ carrier to 10 mmol·g^−1^ carrier increased the specific activity of the immobilized *Ca*LB preparations. A further increase of the amount of bisepoxides to 20 mmol·g^−1^ carrier resulted in negligible increase of activity of the immobilized *Ca*LB compared to that of achievable by *Ca*LB on the carriers treated by 10 mmol bisepoxide g^−1^ carrier. Thus, for both aminoalkyl resins, the optimal amount of bisepoxide for surface modification was 10 mmol·g^−1^ carrier.

**Figure 4:**
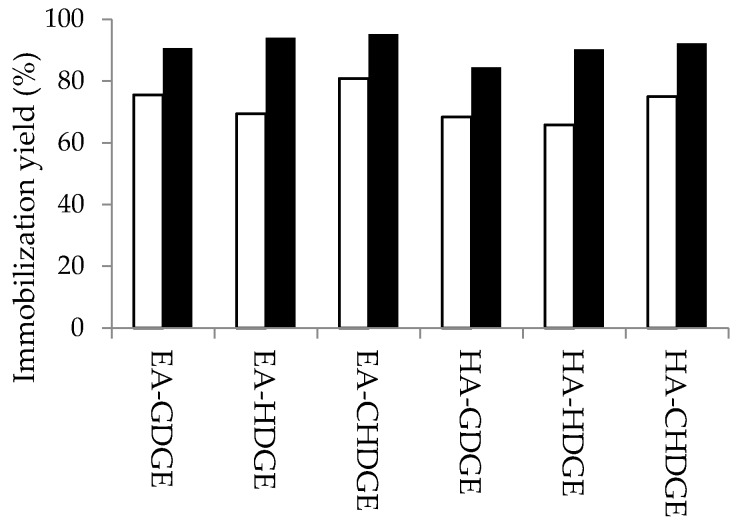
Effect of molar amount of bisepoxide to mass resin ratio on immobilization yields. Supports modified with 2.5 mmol·g^−1^ bisepoxide (□); supports modified with 10 mmol·g^−1^ bisepoxide (■).

This result was confirmed for the other types of bisepoxides by protein concentration assays as well. Immobilization yields for 12 variants of bisepoxide-activated supports were determined by protein concentration measurements in the supernatant before and after immobilization of *Ca*LB according to Bradford’s method. The results for EA- and HA-resins modified with 2.5 and 10 mmol·g^−1^ bisepoxides (CHDGE, HDGE and GDGE) are shown in Figure 4.

Our results indicated that immobilization yields with the other two bisepoxides (CHDGE, HDGE) were also significantly higher after bisepoxide-activation with the optimal amount of bisepoxides (10 mmol·g^−1^ carrier) as compared to the resins modified with only 2.5 mmol bisepoxide g^−1^ carrier. Supports modified with CHDGE showed highest immobilization yields (EA-CHDGE *Ca*LB: 95% and HA-CHDGE *Ca*LB: 92%) which also confirmed the role of proper hydrophobicity of the linker which is beneficial for the first lipase adsorption step during the covalent immobilization on epoxy-activated supports [15].

### 2.4. Operational Stability of the CaLB Preparations

**Figure 5:**
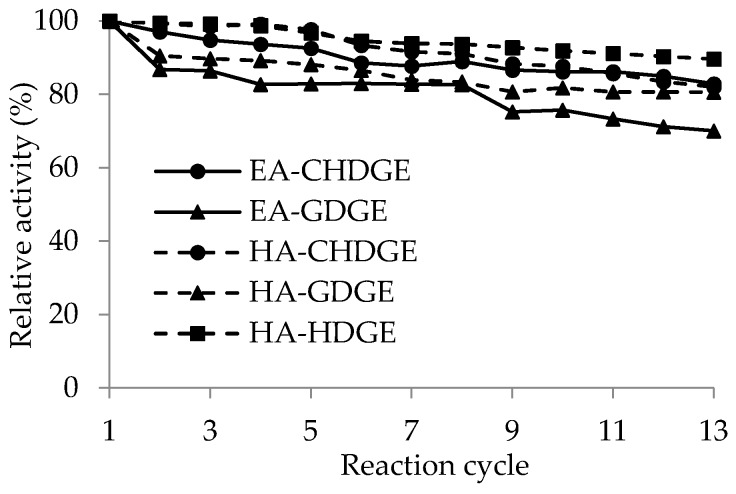
Operational stability of CaLB immobilized on ethylamine (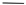) or hexylamine (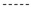) resin activated by CHDGE (●), GDGE (▲) or HDGE (■). Relative activity (%) = activity in given cycle/activity in first cycle × 100 was determined in kinetic resolution of rac-1. Experiments were performed as described in Section 3.9.

One of the great advantages of immobilized enzyme preparations is the possibility of re-use in repeated batch reactions, thus making the process more cost-effective [54]. Five *Ca*LB preparations (EA-GDGE *Ca*LB, EA-CHDGE *Ca*LB, HA-GDGE *Ca*LB, HA-CHDGE *Ca*LB, HA-HDGE *Ca*LB) were selected for studying their stability during recycling. To test their operational stability, the *Ca*LB biocatalysts were tested by repetitive cycles of KRs of *rac-***1** 30 °C for 1 h in a shaken test tube (1000 rpm, in hexane/*t*-butyl methyl ether/vinyl acetate 6/3/1). Between each cycle, the *Ca*LB biocatalysts were washed with *n*-hexane. The relative residual activities of repeated batch experiments compared to the first experiment as 100% are presented in Figure 5. Although every biocatalyst used in this study lost its initial activity cycle-by-cycle, HA-HDGE *Ca*LB retained 90% of its initial activity after 13 cycles. This demonstrated that spacer arms with proper length and hydrophobicity between enzyme molecule and support could contribute not only to the initial activity but also to the long-term stability of the immobilized enzyme. The EA-GDGE *Ca*LB in which the enzyme was attached by the shortest carbon chain retained only 70% of its initial activity after 13 cycles. Furthermore, *Ca*LB immobilized on EA- or HA-resin activated with the cyclohexyl ring containing CHDGE preserved 82% of their initial activity regardless of which resin was used.

### 2.5. Long-Term Storage Stability of the CaLB Preparations

**Figure 6:**
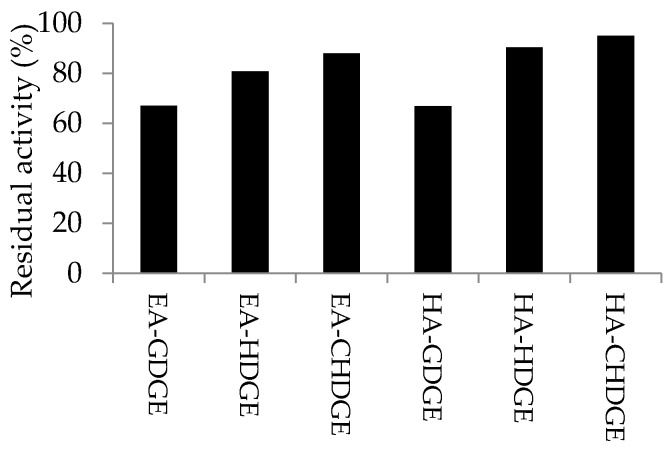
Long-term storage stability of the *Ca*LB biocatalysts. Percentage of the residual activity of the biocatalysts after 12-month storage were compared to the activity of freshly prepared biocatalysts. Experiments were performed as described in Section 3.10.

The long-term storage stability of *Ca*LB preparations were studied by storing the dry biocatalysts in screw capped vials at 4 °C for 12 months. After 12 months, the residual activity of the stored *Ca*LB preparations was compared to the activity of the freshly prepared biocatalysts (results are shown in Figure 6). After 12-month storage, HA-CHDGE *Ca*LB and HA-HDGE *Ca*LB preserved most of their initial activity (95% and 90%, respectively). Presence of the cyclohexyl ring in the linker was beneficial to preserve enzyme activity in case of *Ca*LB attached to modified EA-resin (88% residual activity with EA-CHDGE *Ca*LB), while EA-GDGE *Ca*LB preserved only 67% of its initial activity.

### 2.6. Continuous-Flow Kinetic Resolution of Racemic 1-Phenylethanol (rac-**1**) Catalyzed by CaLB Preparations on Bisepoxide-Activated Resins—Substrate Concentration and Temperature Effects

Immobilization of enzymes and retention of biocatalysts played a major role in development of continuous-flow processes [42,45,46,47,55,56]. It was previously shown that the mode of immobilization [46] and operation temperature [45,50] could significantly influence KR processes in continuous-flow bioreactors. Therefore, six different *Ca*LB preparations from this study were selected to investigate the effect of substrate concentration and temperature on KR of *rac*-**1** catalyzed by the covalently immobilized enzyme. As a reference, this study was extended with the commercially available EP *Ca*LB which contained *Ca*LB covalently immobilized onto epoxy-functionalized acrylic resin.

**Figure 7:**
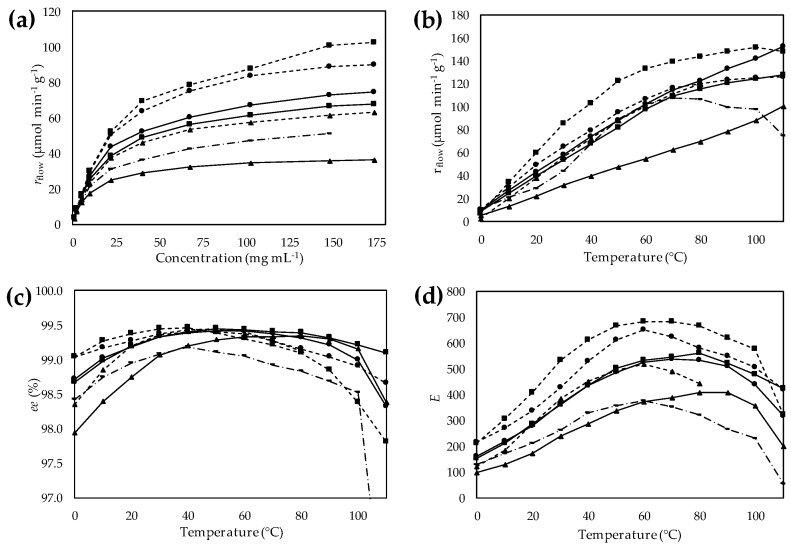
Continuous-flow kinetic resolution of racemic 1-phenylethanol (*rac*-**1**) catalyzed by *Ca*LB preparations on bisepoxide-activated resins. Biocatalytic properties (*r*_flow_, *ee*, *E*) of immobilized *Ca*LB biocatalysts on ethylamine (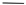) or hexylamine (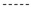) resin activated by CHDGE (●), GDGE (▲) or HDGE (■), compared to EP *Ca*LB (^
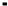
^ in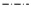). (**a**) Effect of the substrate concentration on specific reaction rate (*r*_flow_); (**b**) Effect of temperature on specific reaction rate (*r*_flow_); (**c**) Effect of temperature on enantiomeric excess (*ee*); (**d**) Effect of temperature on enantiomeric ratio (*E*).

During the substrate concentration dependency investigations, stainless-steel columns filled with the actual immobilized *Ca*LB preparation were thermostated at 30 °C and a solution containing vinyl acetate and the substrate at various concentrations was pumped through the column at a constant flow rate. The productivity of the various *Ca*LB preparations (*r*_flow_, µmol·g^−1^·min^−1^) was investigated as a function of substrate concentration (Figure 7a). The 2.5-fold higher activity of the most active HA-HDGE *Ca*LB than that of the commercially available EP *Ca*LB indicated the significance of fine-tuning the bisepoxide activation for covalent immobilization. Similar to KRs of *rac*-**1** in batch, *Ca*LB on EA- and HA-resins activated with GDGE showed the lowest activity (EA-GDGE *Ca*LB 29.2 µmol·g^−1^·min^−1^; HA-GDGE *Ca*LB 46.1 µmol·g^−1^·min^−1^) among tested derivatives. The behavior of *Ca*LB on resin-surfaces grafted with epoxy function via a linker containing cyclohexyl ring (EA-CHDGE *Ca*LB and HA-CHDGE *Ca*LB derivatives) demonstrated that this lipase interacting with hydrophobic groups became more active in flow systems as well. After an initial quasi-linear range, the productivity–substrate concentration curve showed the typical saturation with all seven *Ca*LB preparations (Figure 7a). The rapidly increasing range of *r*_flow_–[(*R*)-**1**] curves ended at around 40 mg·mL^−1^; therefore, the temperature effect studies were performed at this substrate concentration. 

A major goal of this study was to gain information on the thermal stability of *Ca*LB linked to polymer resins by spacer arms with different length and hydrophobicity. Thus, KR of *rac*-**1** was carried out in bioreactors filled with the selected *Ca*LB preparations similarly as in the substrate-concentration tests but varying the temperature in the range of 0–110 °C in 10 °C steps (Figure 7b–d).

Productivity (*r*_flow_) of the six selected *Ca*LB derivatives on bisepoxide-activated resins as a function of temperature was compared to EP *Ca*LB containing the lipase immobilized on epoxy-functionalized acrylic resin (Figure 7b). The thermal behavior of *Ca*LB attached to bisepoxide activated resins differed significantly from EP *Ca*LB (linking the enzyme to the resin by a quite short linker). The *r*_flow_ of EP *Ca*LB increased only up to 70 °C, then a significant drop of enzyme activity could be observed. In contrast, productivity of *Ca*LB immobilized on EA-resin activated by CHDGE and GDGE bisepoxide increased almost linearly with increasing temperature up to 110 °C indicating the importance of properly selected linkers to achieve significant improvement of thermal stability. The productivity of *Ca*LB immobilized via longer and more flexible linkers (the HDGE-activated EA-resin and the CHDHE- or GDGE-activated HA-resins) increased also uninterruptedly up to 110 °C, although with a slowdown in the increase starting at around 70 °C. HA-HDGE *Ca*LB involving the longest and most flexible linker proved to be the least thermostable of all the six *Ca*LB forms attached to bisepoxide-activated resins with a drop of enzyme activity at 100 °C.

Besides the productivity of the immobilized enzyme, dependence of enantiomer selectivity of the KR (*R*)-**1** on temperature is also an important issue. Thus, enantiomer selectivity of the process was characterized as a function of temperature by the enantiomeric excess of product (*R*)-**1** (*ee*) and by the enantiomeric ratio (*E*) (Figure 7c,d, respectively). The *ee*–temperature (Figure 7**c**) and *E*–temperature (Figure 7d) curves exhibited maxima at various temperatures between 50 and 80 °C. This behavior was in agreement with previously found maxima of *E*–temperature curves in kinetic resolutions of secondary alcohols [45,46,50], although different resins revealed these maxima at different temperatures (Figure 7d). Characteristic differences of enantiomer selectivity optima were found between *Ca*LB on bisepoxide activated EA-resins (~80 °C) compared to *Ca*LB on bisepoxide activated HA-resins (~60 °C) indicating that increased flexibility of the linker allows a lower degree of selectivity. At 60 °C, *Ca*LB immobilized on HA-resin activated by HDGE showed the highest enantiomer selectivity among all tested preparations (*E* ~ 700 with *ee*_(*R*)-**2**_ = 99.3%), while the HA-GDGE *Ca*LB with shorter and less hydrophobic spacer arm showed a lower degree of enantiomer selectivity (*E* ~ 490 with *ee*_(*R*)-**2**_ = 99.2%). On the other hand, *Ca*LB immobilized on EA-resins activated by the three bisepoxides (HDGE, CHDGE and GDGE) displayed maxima in enantioselectivity at around 80 °C (*E* ~ 550, 530 and 380, respectively). This is a remarkable shift compared to the enantiomer selectivity maxima of *Ca*LB immobilized on HA-resins at a 20 °C lower temperature. Worth mentioning is that enantiomer selectivity maxima of both bisepoxide-activated series exceeded the 50 °C optimum found for EP *Ca*LB without a long spacer between the resin surface and the enzyme.

### 2.7. Characterization of Support Morphology after Recycling and Application in Continuous-Flow Reactors

**Figure 8:**
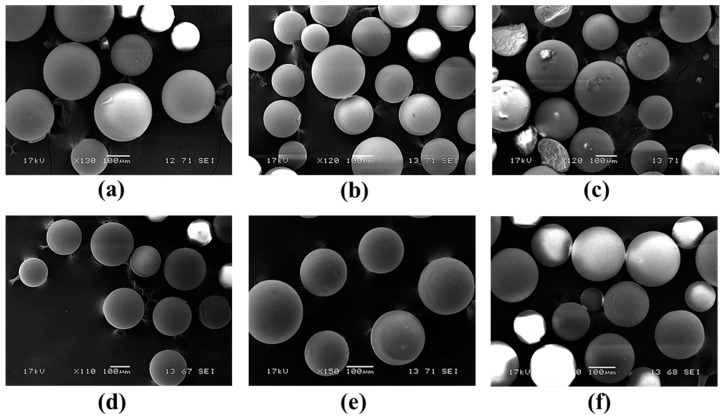
Scanning electron microscopy (SEM) analysis of EA-GDGE *Ca*LB: (**a**) before kinetic resolution of *rac*-**1**; (**b**) after 13 cycles of KRs in batch; (**c**) after the temperature-dependence study in continuous-flow reactor up to 110 °C. Scanning electron microscopy (SEM) analysis of HA-HDGE *Ca*LB: (**d**) before kinetic resolution of *rac*-**1**; (**e**) after 13 cycles of KRs in batch; (**f**) after the temperature-dependence study in continuous-flow reactor up to 110 °C.

In addition to their biocatalytic properties, mechanical stability of the immobilized biocatalysts is also an important issue. Thus, morphology of two selected *Ca*LB forms (on the GDGE-activated EA-resin and on the HDGE-activated HA-resin) representing both aminoalkyl-resin based series was investigated by scanning electron microscopy (SEM) before their use, after the recycling study and after the temperature dependence study (Figure 8).

SEM investigations of EA-GDGE *Ca*LB and HA-HDGE *Ca*LB before their use in biotransformations revealed intact morphology of spherical beads with diameters 150–300 µm (Figure 8a,d, respectively), indicating no mechanical damage during bisepoxide activation and enzyme immobilization steps. Remarkably, both *Ca*LB preparations proved to be mechanically quite durable and remained intact even after 13 cycles of KR experiments in batch mode (Figure 8b,e). However, the SEM pictures after the temperature dependency study of KRs, performed in packed-bed reactors using continuous-flow mode in the 0–110 °C temperature range, indicated limitations in the temperature stability of these *Ca*LB forms on polymer beads (Figure 8c,f). Although data sheets of the basic EA- or HA-resins declared them to be stable up to 60 °C, we tested *Ca*LB on their bisepoxide-activated derivatives up to 110 °C in continuous-flow reactors. Thus, the result indicating fracture of EA-GDGE polymer beads at this elevated temperature was not surprising (Figure 8c). Remarkably, HA-HDGE beads remained apparently unaltered even after the temperature-dependency study ended at 110 °C.

## 3. Materials and Methods 

### 3.1. Materials

Bisepoxides [1,6-hexanediol diglycidyl ether (HDGE), neopentylglycol diglycidyl ether (NDGE), 1,4-cyclohexanedimethanol diglycidyl ether (CHDGE), 1,4-butanediol diglycidyl ether (BDGE), poly(ethylene glycol) diglycidyl ether (PEDGE)] were products of Ipox Chemicals Ltd. (Budapest, Hungary). Racemic 1-phenylethanol (*rac*-1), vinyl acetate, glutaraldehyde (GA) solution (25% w/v in H_2_O); hydrochloric acid, glycerol diglycidyl ether (GDGE) and Triton™ X-100 [4-(1,1,3,3-tetramethylbutyl)phenyl-polyethylene glycol] were purchased from Sigma-Aldrich (Milwaukee, WI, USA). NaH_2_PO_4_, Na_2_HPO_4_ and sodium hydroxide were purchased from Merck KGaA (Darmstadt, Germany).

Solvents (*n*-hexane, *t*-butyl methyl ether, ethanol, Patosolv^®^ (mixture of ethanol/isopropanol, *t*-butanol), propan-1-ol and isopropanol from Molar Chemicals Kft. (Budapest, Hungary) were dried and/or freshly distilled prior to use.

Ethylamine (EA)- and hexylamine (HA)-functionalized methacrylic polymer resins (ReliZyme™ EA 403 and ReliZyme™ HA 403; polymethyl methacrylate supports, particle size 150–300 µm, pore size 400–600 Å) were purchased from Resindion S.r.l. (Rome, Italy).

Lipase B from *Candida antarctica* (*Ca*LB, lyophilized) was purchased from c-LEcta GmbH (Leipzig, Germany). EP *Ca*LB (*Candida antarctica* lipase B, T2-150, covalently attached to dry acrylic beads with a 150–300 µm particle size) was the product of ChiralVision BV (Leiden, The Netherlands).

### 3.2. Analytical Methods

GC analyses were carried out on Agilent 4890 instrument equipped with FID detector using H_2_ carrier gas (injector: 250 °C, FID: 250 °C, head pressure: 12 psi, split ratio: 50:1) on a Hydrodex β-6TBDM column (25 m × 0.25 mm × 0.25 μm film with heptakis-(2,3-di-*O*-methyl-6-*O*-*t*-butyldimethylsilyl)-β-cyclodextrine; Macherey & Nagel GmbH). t_r_ (min): for **1** and **2** (oven: 120 °C, 8 min), 3.62 [(*S*)-**2**], 3.97 [(*R*)-**2**], 5.09 [(*R*)-**1**], 5.35 [(*S*)-**1**]. Conversion (*c*), enantiomeric excess (*ee*) and enantiomeric ratio (*E*) were determined by GC measurements with base-line separations of the enantiomers of **1** and **2** using precise integration methods.

Enantiomeric ratio (*E*) was calculated from the conversion (*c*) and enantiomeric excess of the product (*ee*_(*R*)-**2**_) using the equation *E* = ln[1 − *c*(1 + *ee*_(*R*)-2_)]/ln[1 − *c*(1 − *ee*_(*R*)-2_)] [52]. Due to sensitivity to experimental errors, *E* values calculated in the 100–200 range are given as >100, in the 200–500 range as >200 and above 500 as >>200. 

To characterize the productivity of the biocatalysts, specific reaction rate (or specific biocatalyst activity) in batch reactions before (*U*_b0_) and after (*U*_b_) washing with Triton X-100 solution (for details of washing see Section 3.6) were calculated using the equation *U*_b_ = *n*_(*R*)-**2**_/(*t* × *m*_B_) (where *n*_(*R*)-2_ [μmol] is the amount of the product, *t* [min] is the reaction time and *m*_B_ [g] is the mass of the applied biocatalyst).

Specific reaction rates in continuous-flow systems (*r*_flow_) were calculated using the equation *r*_flow_ = [(*R*)-**2**] × *v*/*m*_B_ (where [(*R*)-**2**] [μmol mL**^−^**^1^] is the molar concentration of the product (*R*)-**2**, *v* [mL·min**^−^**^1^] is the flow rate and *m*_B_ [g] is the mass of the applied biocatalyst) [45].

The surface morphology of the samples was investigated with a JEOL JSM-5500LV scanning electron microscope (SEM). The samples of immobilized *Ca*LB were coated with gold prior to analysis. Electron beam energy of 25 kV was used for the investigations.

### 3.3. Drying of the Aminoalkyl Polymer Resins

The original water content (~60%–70%) of the ethylamine- (EA) and hexylamine-functionalized (HA) beads was removed prior to further use according to the following procedure: In a proper-sized flask, 50.0 g of resins were swirled with ethanol (2 × 100 mL; 2 × 5 min) and hexane (1 × 100 mL; 2 × 5 min), including filtration between the washing steps. After the final filtration, resins were dried at room temperature for 2 h followed by further drying in a VDL 23 vacuum drying chamber, Binder GmbH (Tuttlingen, Germany) at room temperature until the vacuum level dropped below 2 mbar.

### 3.4. Surface Activation of Aminoalkyl Polymer Resins with Bisepoxides or Glutaraldehyde

Dry polymer support (EA- or HA-resin, 500.0 mg) was added to a solution of bisepoxide (2.5 mmol, GDGE, HDGE, NDGE, CHDGE, BDGE or PEDGE) or glutaraldehyde (2.5 mmol) in propan-1-ol (15 mL). The suspension of polymer support in activating agent solution was shaken at 400 rpm for 24 h at 25 °C. The activated support was filtered off on a glass filter (G3), washed with Patosolv^®^ (3 × 10 mL), dried at room temperature (4 h) and stored at 4 °C (in case of the GA-activated support, under argon).

### 3.5. Immobilization of CaLB on Bisepoxide-Activated Polymer Resins

*Ca*LB (40.0 mg, lyophilized powder) was dissolved in phosphate buffer (10.0 mL, 100 mM, pH 7.5), and then the support (200.0 mg) was added to the solution. The enzyme-support suspension was shaken at 400 rpm for 24 h at 25 °C. The immobilized *Ca*LB preparation was filtered off on a glass filter (G3), washed with isopropanol (2 × 15 mL) and hexane (15 mL), dried for 4 h at room temperature and stored at 4 °C. Protein concentration of the *Ca*LB solution before immobilization and in supernatant after immobilization were determined according to Bradford’s method [57]. As a standard, BSA was used.

Immobilization yield (*IY*) was calculated according to equation *IY*(%) = *P/P*_0_ × 100 (where *P*_0_ [mg·mL**^−^**^1^] is the initial protein concentration before immobilization, and *P* [mg·mL**^−^**^1^] is the protein concentration in supernatant after immobilization).

### 3.6. Lipase Desorption Tests

Samples of the immobilized *Ca*LB preparations (80.0 mg of each) were washed at room temperature for 1.5 h with a 1% (*v*/*v*) solution of Triton X-100 in phosphate buffer (7.5 mL; 100 mM; pH 7.5) in a shaker at 400 rpm. The samples were then filtered off on a glass filter (G3) and resuspended in distilled water (15 mL) and incubated for 30 minutes at 400 rpm, filtered off and washed several times with distilled water, isopropanol (2 × 10 mL) and hexane (5 mL).

The “Wash resistance” was calculated using the equation Wash resistance (%) = *U*_b_/*U*_b0_ × 100 (where *U*_b0_ [μmol min**^−^**^1^ g**^−^**^1^] is the specific reaction rate achieved with the immobilized *Ca*LB right after immobilization and *U*_b_ [μmol min**^−^**^1^ g**^−^**^1^] is the specific reaction rate after washing with Triton X-100 solution. For a definition of *U*_b_, see Section 3.2).

### 3.7. Kinetic Resolution of 1-Phenylethanol (rac-**1**) Catalyzed by the CaLB Preparations in Batch Mode

In a screw capped glass vial (4 mL), the immobilized *Ca*LB biocatalyst (25.0 mg) was added to a solution of 1-phenylethanol (*rac*-**1**: 50.0 mg, 0.409 mmol) in a mixture of hexane/t-butyl methyl ether/vinyl acetate 6/3/1 (2.0 mL) and the mixture was shaken (1000 rpm) at 30 °C for 4 h. The reactions were analyzed by GC after 1, 2, and 4 h as described in Section 2.2. All test reactions were performed in triplicates. Standard deviations of conversion were below 5%, standard deviations of enantiomeric excess were below 0.4%.

### 3.8. Optimization of the Bisepoxide Activation of Alkylamino Polymer Resins

Bisepoxide (GDGE, HDGE or CHDGE) in various molar amounts (1.25 mmol, 2.5 mmol, 5 mmol or 10 mmol) was dissolved in propan-1-ol (15 mL) and then polymer support (EA or HA, 500.0 mg) was added to the solution. The mixture was shaken (400 rpm) at 25 °C for 24 h. The bisepoxide-activated support was filtered off on a glass filter (G3), washed with Patosolv^®^ (3 × 10 mL), dried at room temperature (4 h) and stored at 4 °C. Catalytic properties of the *Ca*LB on the bisepoxide-activated support was tested (for details of immobilization, washing and testing see Section 3.5, Section 3.6 and Section 3.7, respectively).

### 3.9. Recycling of the Immobilized CaLB Biocatalysts

Immobilized *Ca*LB biocatalyst (25.0 mg) was added to a solution of 1-phenylethanol (*rac*-**1**, 50.0 mg, 0.409 mmol) in hexane/*t*-butyl methyl ether/vinyl acetate 6/3/1 (1.0 mL) in an Eppendorf tube and the mixture was shaken (1000 rpm) for 1 h at 30 °C. After 1 h, the reaction mixture was centrifuged, the immobilized *Ca*LB biocatalyst was washed with hexane (2 × 1.0 mL), then fresh solution of *rac*-**1** (50.0 mg, 0.409 mmol) in hexane/*t*-butyl methyl ether/vinyl acetate 6/3/1 (1.0 mL) was added to biocatalysts and samples were shaken again (1000 rpm) at 30 °C for 1 h. In this way, immobilized *Ca*LB biocatalysts were tested in 13 consecutive cycles.

### 3.10. Long-Term Stability of CaLB Derivatives (Storage at 4 °C)

Residual activities of enzyme preparations (EA-GDGE *Ca*LB, EA-HDGE *Ca*LB, EA-CHDGE *Ca*LB, HA-GDGE *Ca*LB, HA-HDGE *Ca*LB, HA-CHDGE *Ca*LB) stored at 4 °C in glass vials closed with screw caps under argon for 12 months in kinetic resolution of *rac*-**1** were determined as described in Section 3.7.

### 3.11. Packed-Bed Columns Filled with Immobilized CaLB

Immobilized *Ca*LB preparations [EP *Ca*LB, EA-GDGE *Ca*LB, EA-HDGE *Ca*LB, EA-CHDGE *Ca*LB, HA-GDGE *Ca*LB, HA-HDGE *Ca*LB, HA-CHDGE *Ca*LB) were packed into stainless steel CatCart™ columns (stainless steel, inner diameter: 4 mm; total length: 70 mm; packed length: 65 mm; inner volume: 0.816 mL) according to the filling process of ThalesNano Inc (Budapest, Hungary). The columns were settled by silver metal [Sterlitech Silver Membrane from Sigma-Aldrich (Milwaukee, WI, USA), Z623237, pore size 0.45 µm; purity: 99.97%] and PTFE [Whatman^®^ Sigma-Aldrich, Milwaukee, WI, USA, WHA10411311, pore size 0.45 µm] filter membranes. Two CatCart™ columns per enzyme preparation (separate columns for temperature and substrate concentration test) were packed (filling weights: EP *Ca*LB, 250 ± 10 mg; HA-GDGE *Ca*LB, 230 ± 13 mg; HA-HDGE *Ca*LB, 220 ± 2 mg; HA-CHDGE *Ca*LB, 230 ± 15 mg; EA-GDGE *Ca*LB, 229 ± 5 mg; EA-HDGE *Ca*LB, 212 ± 9 mg; EA-CHDGE *Ca*LB, 214 ± 14 mg).

### 3.12. Kinetic Resolution of 1-Phenylethanol (rac-**1**) Catalyzed by the CaLB Preparations in Continuous-Flow Mode

Continuous-flow kinetic resolutions were performed in a laboratory flow reactor built from a Knauer K-120 isocratic HPLC pump attached to CatCart™columns filled with the immobilized *Ca*LB biocatalysts in an in–house made aluminum metal block column holder with precise temperature control. The *Ca*LB-filled columns were washed with a dry hexane/*t*-butyl methyl ether 2/1 mixture (1 mL·min**^−^**^1^, 20 min) before reactions with *rac*-**1**.

To study the effect of substrate concentration on the biocatalytic properties of immobilized *Ca*LB biocatalysts, the solution of 1-phenylethanol (*rac*-**1**) at different concentrations (1, 2.5, 5, 9.5, 22, 40, 68, 103, 148, 174 mg·mL**^−^**^1^) and vinyl acetate (5 equiv.) in dry hexane/*t*-butyl methyl ether 2/1 was pumped through the enzyme-filled columns set to 30 °C at a flow rate of 0.2 mL·min**^−^**^1^. Samples were analyzed by GC every 10 min up to 40 min to allow the establishment of a stationary state. Samples were collected during stationary operation (40 min after changing the parameters) and analyzed as described in Section 3.2.

### 3.13. Effect of Temperature on Kinetic Resolution of 1-Phenylethanol (rac-**1**) Catalyzed by the CaLB Preparations in Continuous-Flow Mode

The solution of 1-phenylethanol *rac*-**1** (40.0 mg·mL**^−^**^1^) and vinyl acetate (5 equiv.) in hexane/*t*-butyl methyl ether 2/1 was pumped through the biocatalyst-filled columns thermostated to various temperatures (0–110 °C) at a flow rate of 0.2 mL·min**^−^**^1^. Samples were collected during stationary operation (40 min after changing the parameters) and analyzed as described above. The experiments were performed with 10 °C steps in a temperature range between 0 and 110 °C. 

After completing experiments at different substrate concentrations and temperatures, the columns were routinely washed with hexane/*t*-butyl methyl ether 2/1 mixture (0.5 mL·min**^−^**^1^, 20 min) and then analyzed by scanning electron microscope (for details on SEM, see Section 3.2).

## 4. Conclusions

Covalent immobilization of *Candida antarctica* lipase B (*Ca*LB) was successfully carried out on bisepoxide-activated aminoalkyl polymer resins. Our study with six bisepoxides of different chemical features as activating agents for macroporous acrylate resins with ethylamine and hexylamine functions on their surface revealed that, by selecting the proper bisepoxide activation, tuning of the biocatalytic properties of the immobilized *Ca*LB was feasible. Re-use and long-term stability studies indicated that bisepoxide-activated polymer supports provided a suitable micro-environment for *Ca*LB molecule during catalysis and storage. Bisepoxide 1,4-cyclohexanedimethanol diglycidyl ether—CHDGE proved to be especially suitable in activating alkylamino resins for covalent immobilization of CaLB by providing a hydrophobic surface and epoxy function suitable for covalent immobilization in a single step. The thermal stability of covalently immobilized biocatalysts was studied between 0 and 110 °C in continuous-flow packed-bed reactors and was compared to commercially available *Ca*LB immobilized on macroporous acrylic epoxy resin. Spacer arms of various lengths, hydrophobicity and rigidity markedly altered the biocatalytic properties of *Ca*LB attached to polymer resins. It seemed that hydrophobic stabilization of the immobilized enzyme by a relatively rigid and hydrophobic spacer arm contributed significantly to preservation of enzyme activity and selectivity at higher temperatures.

## Figures and Tables

**Table 1:** Properties of polymer resins with aminoalkyl functions applied for *Ca*LB immobilization.

Polymer Resin	Resin Name (Abbreviation)	Pore Size (nm)	Particle Size (µm)	Ion Exchange Capacity, Wet (µmol·g^−1^)	Water Retention (%)
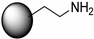	ReliZyme™ EA 403 (EA)	40–60	100–300	500	60–70
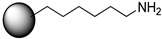	ReliZyme™ HA 403 (HA)	40–60	100–300	600	60–70

**Table 2:** Kinetic resolution of 1-phenylethanol (*rac*-**1**) catalyzed by *Ca*LB on bisepoxide-activated polymer resins (molar amount of bisepoxide 5 mmol·g*^−^*^1^ carrier) compared to the catalysis by *Ca*LB on a commercial epoxy resin, on unmodified and on GA activated EA-resins. Results with the various forms of *Ca*LB after washing with Triton X-100 are shown. Experiments were performed with preparations at 30 °C for 2 h, as described in Section 3.7.

Binding Function	Wash Resistance ^a^ (%)	*U*_b_ (µmol·min^−1^·g^−1^)	*c* (%)	*ee*_(*R*)-2_ (%)	*E* ^b^
EP ^c^	^d^	39.6	28.9	99.1	>200
EA ^e^	7	0.4	0.3	^f^	^f^
EA-GA	39	10.2	7.4	98.9	>200
EA-GDGE	53	23.7	17.4	99.4	»200
EA-BDGE	22	8.5	6.2	98.2	>200
EA-HDGE	58	27.5	20.2	99.4	>200
EA-CHDGE	81	39.6	28.9	99.5	»200
EA-PEDGE	7	2.2	1.6	^f^	^f^
EA-NDGE	30	12.0	8.8	98.6	>200
HA ^g^	11	0.2	0.2	^f^	^f^
HA-GA	51	11.9	8.7	98.9	>200
HA-GDGE	64	23.8	17.4	99.6	»200
HA-BDGE	51	24.5	17.9	99.2	»200
HA-HDGE	82	44.2	32.4	99.5	»200
HA-CHDGE	92	47.6	34.8	99.4	»200
HA-PEDGE	17	6.3	4.6	^f^	^f^
HA-NDGE	49	20.2	14.7	99.1	>200

^a^ Wash resistance (%) = activity of samples washed with Triton X-100/activity of samples after immobilization × 100, in kinetic resolution of *rac*-**1**; ^b^ Enantiomeric ratio [52]; ^c^ EP *Ca*LB: *Ca*LB immobilized onto epoxy-functionalized acrylic beads; ^d^ Activity of EP *Ca*LB washed with Triton X-100/activity of EP *Ca*LB × 100 = 125%; ^e^ Ethylamine resin without activation; ^f^ *ee* and *E* values are not given for reactions with *c* < 5% due to their high uncertainty; ^g^ Hexylamine resin without activation.

## Abbreviations

The following abbreviations are used in this manuscript:

*Ca*LB	*Candida antarctica* lipase B
EA	ethylamine functionalized macroporous acrylate resin
HA	hexamethylamine functionalized macroporous acrylate resin
GDGE	glycerol diglycidyl ether
HDGE	1,6-hexanediol diglycidyl ether
CHDGE	cyclohexanedimethanol diglycidyl ether
BDGE	1,4-butanediol diglycidyl ether
NDGE	neopentylglycol diglycidyl ether
PEDGE	polyethyleneglycol diglycidyl ether
GA	glutaraldehyde
